# Phosphoryl-Graphene for High-Efficiency Uranium Separation and Recycling

**DOI:** 10.1021/acsami.4c22385

**Published:** 2025-02-20

**Authors:** Martin Pykal, Veronika Šedajová, Aditya Thakur, Somnath Sengupta, Cherukuri Venkata Siva Brahmananda Rao, Radek Zbořil, Balija Sreenivasulu, Michal Otyepka, Kolleboyina Jayaramulu

**Affiliations:** † Regional Centre of Advanced Technologies and Materials, Czech Advanced Technology and Research Institute (CATRIN), Palacký University Olomouc, Šlechtitelů 27, Olomouc 779 00, Czech Republic; ‡ Hybrid Porous Materials Laboratory, Department of Chemistry, Indian Institute of Technology Jammu, Jammu and Kashmir 181221, India; § Fuel Chemistry Division, Materials Chemistry and Metal Fuel Cycle Group, Indira Gandhi Centre for Atomic Research, Kalpakkam 603102, India; ∥ Homi Bhabha National Institute, Anushaktinagar, Mumbai 400094, India; ⊥ Nanotechnology Centre, Centre for Energy and Environmental Technologies, VŠB − Technical University of Ostrava, 17. Listopadu, Ostrava-Poruba 708 00, Czech Republic; # IT4Innovations, VŠB-Technical University of Ostrava, 17. listopadu 2172/15, Ostrava-Poruba 70800, Czech Republic

**Keywords:** two-dimensional (2D) materials, phosphoryl-functionalized graphene, graphene derivatives, uranium adsorption, molecular dynamics simulations

## Abstract

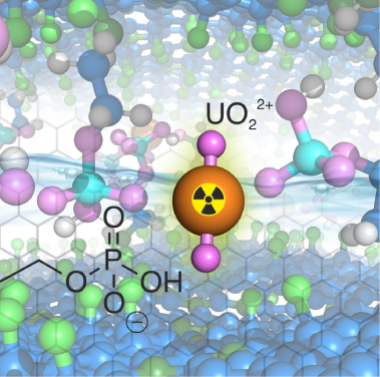

To enhance the sustainability of nuclear energy and protect the environment, the efficient extraction of uranium from various water sources has emerged as an essential strategy for addressing the long-term challenges of nuclear waste management. In this study, we designed phosphoryl-functionalized graphene (PG) for efficient uranyl adsorption and synthesized the material from fluorinated graphene using phosphoryl ethanolamine under solvothermal conditions. The resultant PG features a unique 2D structure equipped with solvent-exposed phosphoryl groups, making it highly suitable for uranium adsorption in aqueous solutions. Notably, PG demonstrated a high sorption efficiency (∼77%) with rapid extraction capability (∼5 min) for U(VI) from aqueous media at pH 7, achieving an adsorption capacity of 316 mg U g^–1^. It also demonstrates good recyclability and stability even after 3 cycles and exhibits a significant seawater adsorption capacity of 117.8 mg U g^–1^. Both X-ray photoelectron spectroscopy analysis and molecular dynamics simulations revealed a preferential binding of uranyl ions to the phosphoryl groups of PG. This work paves the way for designing and developing functional graphene derivatives for efficient uranium extraction from various water resources, with promising potential for the recovery of other radioactive elements.

## 1 Introduction

Nuclear energy is essential for lowering carbon emissions while supporting long-term economic growth and environmental sustainability.^1^ As uranium is essential for powering nuclear reactors, the industry faces challenges due to limited terrestrial reserves.^2^ Seawater, however, contains an estimated 4.5 billion tons of uranium, representing a substantial potential resource for uranium. Despite the presence of uranium in seawater, its extraction is difficult because of its low concentration of approximately 3.3 ppb and the complex composition of seawater. Major obstacles persist in developing materials and techniques to enhance uranium recovery from seawater.^3−5^ The growing demand for uranium recovery has prompted recent research into chemical methods for removing U(VI) from water, such as solvent extraction, coprecipitation, ion exchange, and adsorption.^6^ However, the adsorption method, which utilizes conventional porous adsorbents like porous organic polymers, covalent organic frameworks, porous aromatic frameworks, metal–organic frameworks, and siderophore-inspired chelators, is particularly notable for its cost-effectiveness, operational simplicity, and high efficiency, especially in capturing trace amounts of uranium. Despite the suggested good adsorption capacity of porous materials, they have limitations such as low adsorption capacity and poor regeneration ability.^7−13^ Additionally, these porous adsorbents are unstable under acidic/basic conditions and lack selective adsorption for uranium, further limiting their practical applications.^14^

**Scheme 1:**
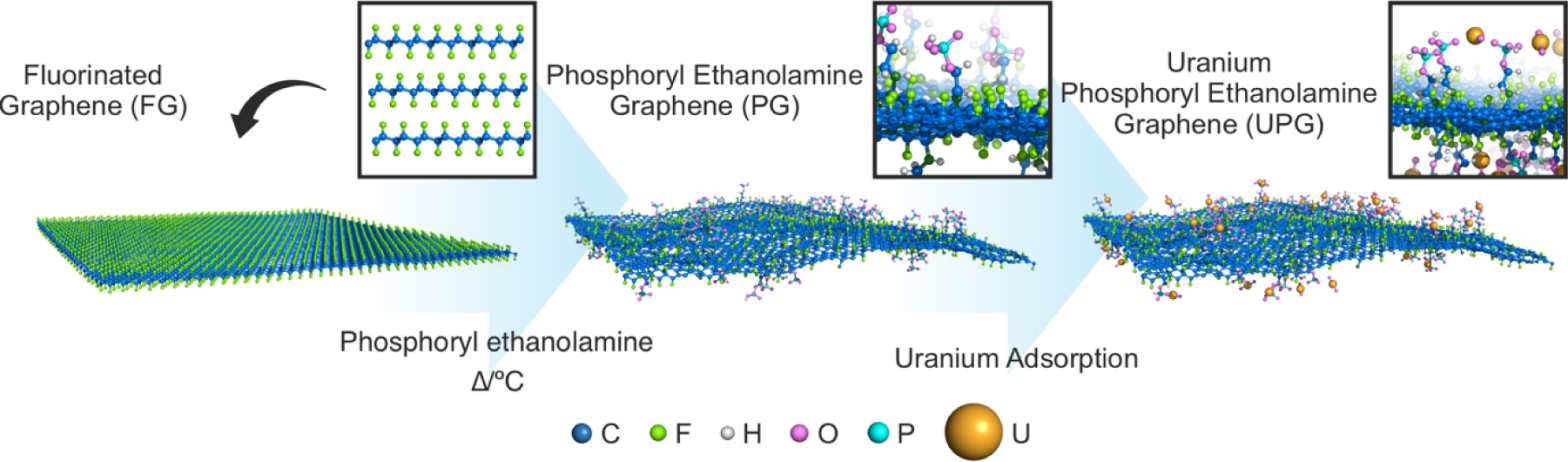
Schematic Illustration Depicting the Solvothermal Synthesis of Phosphoryl-Functionalized Graphene (PG) from FG The resulting PG demonstrates enhanced uranium extraction capabilities from aqueous water solutions.

Recently, two-dimensional (2D) materials like graphene, graphene oxide (GO), MXenes, boron nitride (BN) and some transition metal dichalcogenides such as MoS_2_, have been used for water purification applications owing to their unique physicochemical properties, including atomic thickness, a large aspect ratio, chemical flexibility, and abundant functional groups.^15−20^ There are two types of nanopores in 2D materials: (i) interlayer/intralayer nanochannels formed by stacking of 2D nanosheets, and (ii) nanoscale pores within the nanosheets, which^21^ occur as defects in atomically thick 2D nanosheets during synthesis.^21−25^ Furthermore, the atomic thinness of 2D materials lowers resistance to mass transport in water separation applications.^26−28^ However, challenges remain, such as the difficulty in creating uniform and well-dispersed nanopores, along with the high cost of drilling, which hampers the advancement of 2D materials in this field.^29^ Within the family of 2D materials, GO and reduced GO (RGO) have attracted significant interest for water purification and wastewater treatment because of their exceptional characteristics, including outstanding dispersion properties, hydrophilicity, and compatibility.^30,31^ GO and RGO derivatives can coordinate uranium from water because of reactive, oxygen-containing groups on their surfaces.^27,32,33^ However, their effectiveness has been limited because the functional groups are mostly situated at the edges rather than on the flat surface. To overcome challenges in uranium extraction, it is essential to systematically introduce specific chemical groups that can effectively interact with uranium-containing chemicals and that are evenly distributed across the graphene surface. Employing fluorographene (FG) chemistry is highly attractive for this purpose because FG functionalization results in homogeneously surface-functionalized graphene derivatives.^34−36^ The versatility of FG chemistry offers the mounting of various chemical moieties, which can also be effectively used for ion separation technologies, including energy storage/harvesting applications.^37,38^ Introducing pendant anionic phosphoryl functional groups onto graphene surfaces can represent an effective strategy for immobilization and migration control of U(VI) ions. Therefore, grafting phosphoryl groups onto graphene surfaces seems to be a rational strategy for designing 2D carbon-based materials efficient for uranium removal. Herein, we synthesized phosphoryl-functionalized graphene (PG) on the basal plane by strategically converting the fluorine groups of FG to phosphoryl ethanolamine moieties under solvothermal conditions, as shown in Scheme 1. The resultant PG shows maximum adsorption of uranium with a sorption efficiency of ∼77% due to the 2D structure with homogeneous functionalization with phosphoryl groups. The synthesized PG exhibited remarkably rapid extraction capabilities (∼5 min) for U(VI) from aqueous solutions, achieving an extraction efficiency of approximately 316 U g^–1^ at pH 7. Notably, the reported sorption capacities significantly exceeded those of other 2D sorption materials. It is worth highlighting that binding of U(VI) to the adsorption surface of PG can cause considerable variations in adsorption potential and binding energies. The bonding mechanism between U(VI) and PG was further validated through all-atom molecular dynamics (MD) simulations in water and X-ray photoelectron spectroscopy (XPS). This study provides a basis for engineering functionalized graphene derivatives with significantly improved stability characteristics. These advanced 2D materials show promise for the rapid and high-yielding removal of U(VI) from pH-specific environments, particularly from seawater and mine water, for applications in the nuclear industry.

## 2 Experimental Section

### 2.1 Materials

Graphite fluoride (>61 wt % F), *O*-Phosphorylethanolamine (*O*-PEA, ≥98%) and *N*,*N*-Dimethylformamide (DMF, ≥98%) were acquired from Sigma-Aldrich, while pure acetone and absolute ethanol came from Penta, Czech Republic. All aqueous solutions were prepared using ultrapure water (18 MΩ cm).

### 2.2 Synthesis of Phosphoryl Functionalized Graphene (PG)

Graphite fluoride (0.5 g) was dispersed in 30 mL of DMF within a glass flask. The resulting mixture was stirred at 600 rpm using a Teflon-coated magnetic stirrer for a period of 3 days. Following the stirring period, the mixture was subjected to sonication using a Bandelin Sonorex (DT255H type, 35 kHz, 160 W) for 4 h, and subsequently stirred overnight. Subsequently, 1.5 g of o-PEA was added. Then, the dispersion was heated to 130 °C for 3 days using a condenser with continuous stirring at 600 rpm using an oil bath for heating. Post-reaction, the mixture was cooled and the product was thoroughly washed with DMF, acetone, ethanol, and finally water. Centrifugation (Sigma 4-16K, 13,000 rcf) was employed to separate the solid, and washing with water continued until no further precipitation occurred during centrifugation.

### 2.3 U(VI) Sorption Studies

Sorption studies of U(VI) were conducted using PG across a wide range of pH using batch techniques. A U(VI) stock solution (∼2 mg mL^–1^ or 2000 ppm) was utilized to prepare solutions at various pH levels and concentrations. Aqueous solutions with pH values ranging from 2 to 9 were created by diluting stock solution aliquots to 500 ppm within 25 mL volumetric flasks, with the entire process conducted at room temperature. Small quantities of 0.1 M HNO_3_, 3 M NaOH, and deionized water were employed to adjust the pH to the desired values. Subsequently, 10 mg of PG was placed in equilibration tubes, followed by the addition of 3 mL of the prepared pH solutions containing U(VI) at a concentration of 500 ppm. Following a 3-h equilibration period, the solutions were centrifuged at 7000 rpm for 15 min, after which the supernatant was collected. The presence of U(VI) was determined using UV–vis spectrophotometry. A colorimetric analysis was performed utilizing Arsenazo-III as the chromogenic indicator for U(VI) detection, exhibiting maximum absorbance (λ_max_) at approximately 655 nm. The sorption efficiency (%), the amount of metal ion taken up by the PG (*q*_e_; mg g^–1^) and the distribution coefficient (*K*_d_) for U(VI) adsorption by PG were calculated using eqs S1–S3.

### 2.4 Kinetics of Adsorption

Kinetic studies were conducted to elucidate the underlying mechanism of U(VI) adsorption onto PG. Pseudo-first-order and pseudo-second-order kinetic models were applied to the experimental data to determine the best-fitting model and its associated rate constants. Detailed kinetic equations and parameter definitions are provided in the Supporting Information.

### 2.5 Adsorption Isotherms

Adsorption isotherms were employed to characterize the interactions between U(VI) and PG. The characteristics of the adsorption process were elucidated by fitting the experimental data to the Langmuir and Freundlich isotherm models. Detailed isotherm equations and parameter definitions are available in the Supporting Information.

### 2.6 Thermodynamic Parameters

The thermodynamic favorability of U(VI) adsorption onto PG was assessed by calculating the thermodynamic parameters Δ*H*, Δ*S*, and Δ*G*. The calculations were performed at different temperatures, and the Van’t Hoff plot was used to obtain Δ*H* and Δ*S* from its slope and intercept, respectively. The equations used for calculating thermodynamic parameters are presented in the Supporting Information.

### 2.7 Desorption and Recyclability Studies

Desorption studies were conducted to assess the release of previously adsorbed U(VI) from PG. Various eluents were used in the desorption studies, including deionized water, 0.01 M HNO_3_, 0.1 M HNO_3_, 0.01 M Na_2_CO_3_, and 0.1 M Na_2_CO_3_. For 2 h, a PG sample (approximately 10 mg) was equilibrated with 3 mL of each eluent. Equilibrated solutions were separated by centrifugation, and the resulting supernatant was analyzed for U(VI) using UV spectrophotometry. Recycling studies were conducted to determine the reusability of PG for U(VI) sorption. Three mL of U(VI) (300 mg L^–1^) solution was mixed with 10 mg of PG and equilibrated for 30 min at room temperature. Following equilibration, the solutions were centrifuged, and the supernatant was removed for residual U(VI) analysis. The filtered material was extensively cleaned with deionized water and dried at 100 °C for 24 h. Following this, the loaded U(VI) on PG was removed using the desorption technique. The recovered powder could then be utilized for U(VI) sorption for two to three rounds of recyclability testing. The powder XRD analysis was also employed to investigate the structural retainability of PG following recyclability tests. Here, recyclability studies were performed two times for U(VI). The extent of removal of uranyl ions (R%) was calculated from the following eq 1:
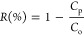
1where *C*_0_ and *C*_p_ are concentrations of uranium before and after adsorption, respectively.

### 2.8 Capture Study of Uranium with Other Metal-Ions

The effect of interfering ions on uranium adsorption was tested in experiments with equimolar amounts of individual and combined salts (KCl, NaCl, CaCl_2_, Mg(NO_3_)_2_, SrCl_2_, CdCl_2_). Adsorption studies were performed using 2 mg of adsorbent and 2 mL of uranium-spiked water (initial U(VI) concentration of 5 ppm). These mixtures were stirred vigorously for 2 h. After 12 h, the filtrate was collected (0.2 μm syringe filter) and analyzed for U(VI) using EDS.

### 2.9 Molecular Dynamic Simulations

MD simulations were conducted employing the GROMACS 2023 software package with the OPLS-AA force field.^39,40^ U(VI) ions were modeled using parameters from Zhang et al.,^41^ originally derived by Greathouse et al.^42^ The parameters for the fluorographene surface, which was represented as a periodic 10 × 10 nm sheet, originated from OPLS-AA for per-fluorinated molecules.^43^ In our model, a total of 76 phosphoryl ethanolamine groups were randomly attached uniformly across both sides of the surface. To simulate different pH environments, two phosphate protonation states were considered, either −1*e* or −2*e*. The partial charges of the functional phosphoryl ethanolamine groups were derived using the Restrained Electrostatic Potential (RESP) procedure on a small functionalized pyrene system.^44^ Due to the limited number of ions, the concentration of U(VI) was increased to 0.2 M, representing 64 ions. The net charge was neutralized depending on the protonation state of phosphate groups either with Cl^–^ or Na^+^ ions using the Joung-Cheatham ion parameters.^45^ 21% of the carbon atoms in the lattice exhibited sp^3^ hybridization, incorporating residual fluorine atoms. Additionally, the surface was doped with 5% graphitic nitrogen. The resulting simulation box, with dimensions of 100 × 100 × 50 Å, was filled with SPC/E water molecules.^46^ Simulations were performed with periodic boundary conditions applied in all three dimensions. Interatomic interactions were modeled using the Lennard-Jones 12–6 potential, truncated at a cutoff distance of 10 Å.^47^ Hydrogen atoms were constrained using the LINCS algorithm. The equations of motion were integrated using a time step of 2 fs. Prior to production, the system underwent energy minimization followed by a 5 ns thermalization phase, where the temperature was gradually increased from 10 to 300 K using the V-rescale thermostat^48^ (coupling constant of 0.1 ps). Subsequently, the system was equilibrated in the NpT ensemble at 300 K for an additional 5 ns, employing the Berendsen barostat^49^ with a reference pressure of 1 bar and a coupling constant of 1.0 ps. The production run was conducted for 100 ns, with trajectory data saved every 2 ps. Visual representations of the simulation results were generated using PyMOL (The PyMOL Molecular Graphics System, Version 3.0 Schrödinger, LLC).

## 3 Results and Discussion

### 3.1 Synthesis and Characterization of PG

Our group successfully synthesized various functional graphene derivatives using FG, where fluorine groups are transformed into selective functional groups.^50^ Herein, we synthesized phosphoryl-functionalized graphene (PG) using scalable and controllable FG chemistry.

**Figure 1:**
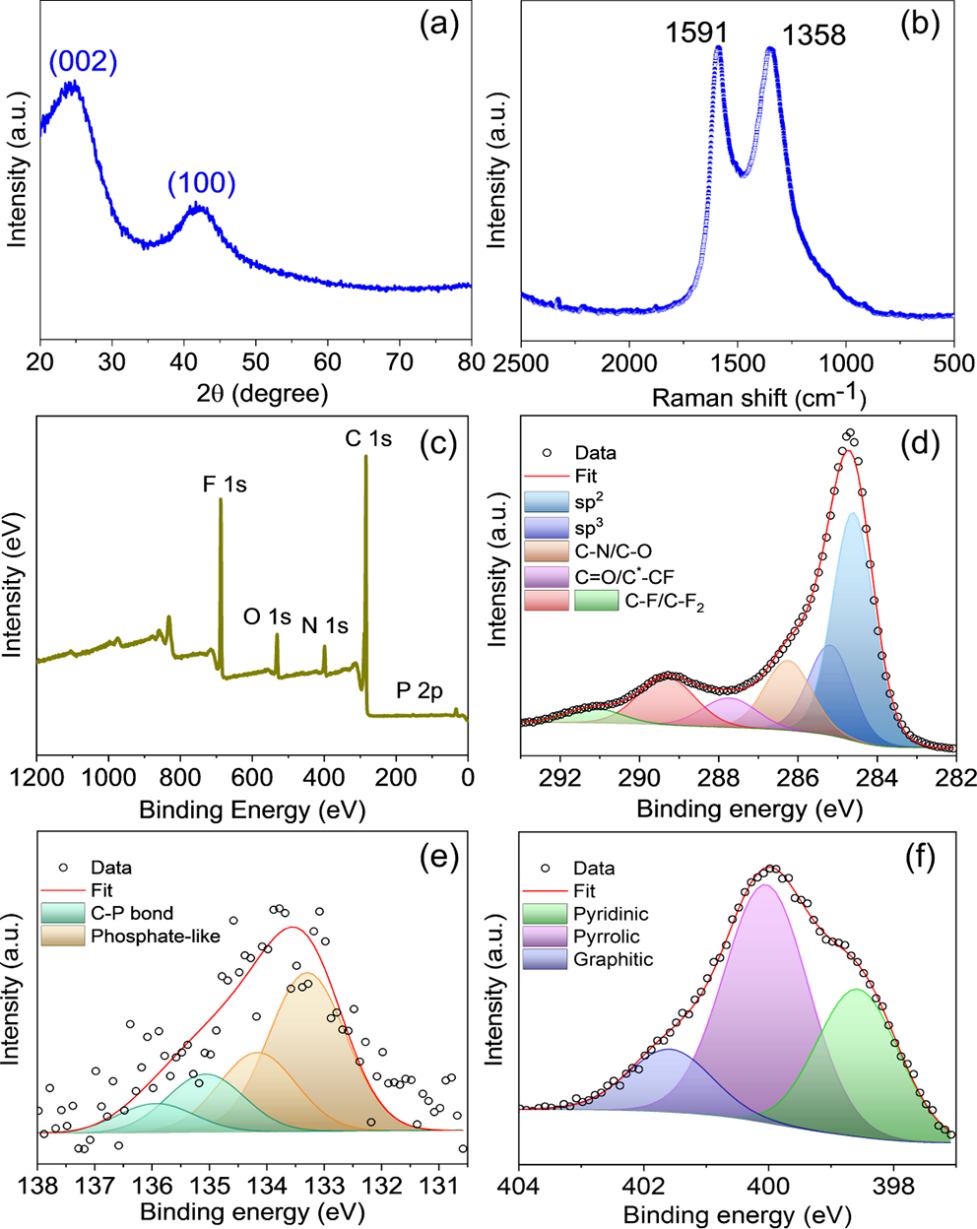
(a) PG powder XRD pattern; (b) Raman spectrum; (c) survey spectrum; and high-resolution XPS spectra of (d) C 1s, (e) P 2p, and (f) N 1s regions, respectively.

Phosphoryl-modified graphene (PG) was prepared from exfoliated graphite fluoride, i.e., FG, by reacting it with phosphoryl ethanolamine under solvothermal conditions, as illustrated in schematic representation (Scheme 1). Powder X-ray diffraction (PXRD) was employed to assess the structure and phase purity of the synthesized material revealing two peaks at 24.5° and 42.7°, attributed to the (002) and (100) reflections, respectively (Figure 1a). Fourier-transform infrared spectroscopy (FT-IR) indicates peaks at 1200 and 1305 cm^–1^ in FG, attributed to CF and CF_2_ groups, whereas the new bands at around 1106 and 1180 cm^–1^ in PG, can be attributed to the P–O, C−C, and P=O bonds of phosphoryl ethanolamine modified graphene (Figure S1). Raman spectroscopy of PG revealed characteristic D and G bands at 1358 and 1591 cm^–1^, respectively. The calculated *I*_D_/*I*_G_ ratio was 0.85 (Figure 1b). The broadness of the D-band indicates the presence of a significant number of sp^3^ carbons and defects associated with the P, O and remaining fluorine functional groups, which are created during the chemical treatment. Further, the XPS survey spectrum confirms the presence of C, O, N, F, and P in the PG sample, with atomic percentages of 73.8, 5.4, 3.6, 14.7, and 0.11 at. %, respectively (Figure 1c), yielding the atomic percentage of the functional groups of 0.8%. Deconvolution of the high-resolution C 1s spectrum revealed six distinct components, corresponding to various carbon bonding configurations: C–C (sp^2^), C–C (sp^3^), carbon bonded to nitrogen and oxygen and unreacted fluorine moieties (Figure 1d). The high-resolution P 2p was deconvoluted into two doublets with their typical spin–orbit splitting of 0.87 eV.^51^ These two doublets slightly overlap and correspond to a phosphate-like phosphorus environment and to a carbon–phosphorus bond, respectively, as reported in the literature (Figure 1e).^52−54^

**Figure 2:**
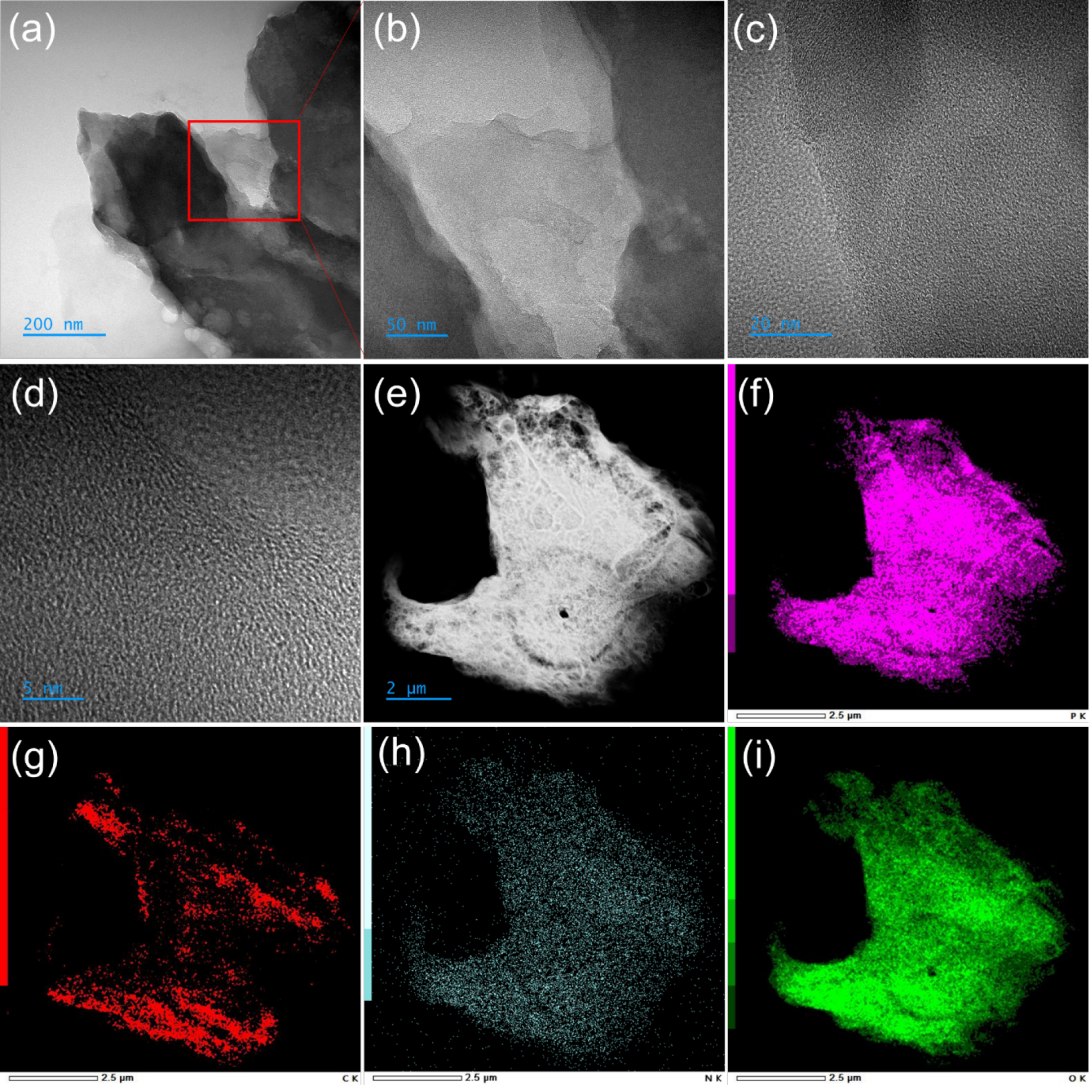
Microscopic analysis of PG (a–d). HRTEM image of PG shows its layered nature with different magnifications. Elemental mapping indicates a uniform distribution of monitored elements. (e) HAADF image, (f) phosphorus in purple, (g) carbon in red, (h) nitrogen in cyan, and (i) oxygen in green.

Further, in the high-resolution N 1s spectrum, three peaks were deconvoluted and attributed to three common types of nitrogen–pyridinic, pyrrolic and graphitic–which are found in derivatives of FG, treated in DMF (Figure 1f).^55^ The HRTEM analysis of PG at various magnifications reveals a bundle of randomly organized nanosheets interspersed with irregularly arranged features at different depths (Figure 2a–d). Moreover, a typical HAADF-TEM image of the PG confirmed the presence of C, N, O, and P, distributed homogeneously within the sample (Figure 2e-i). The XPS, Raman, and powder XRD analyses reveal that the PG possesses phosphoryl functional groups on the basal plane.

### 3.2 Molecular Dynamics (MD) Simulation Studies

**Figure 3:**
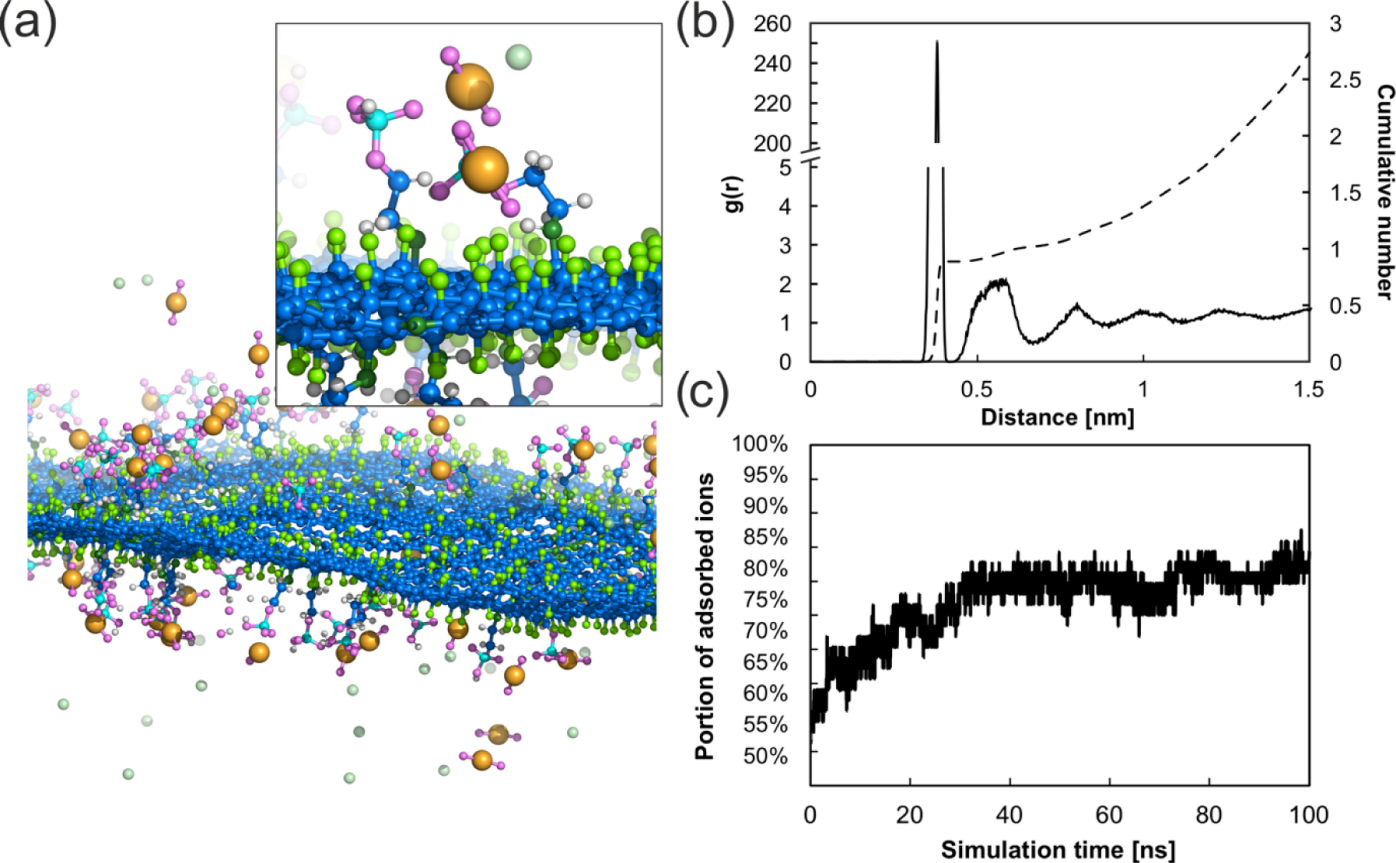
(a) Snapshot from MD simulation showing a structure of the PG material with detail of interacting phosphoryl ethanolamine groups with U(VI) ions. (b) Radial distribution function and corresponding cumulative number of U(VI) around P atom. (c) Percentage of adsorbed U(VI) ions on the PG surface. Color code: oxygen, pink; nitrogen, dark green; carbon, blue; phosphorus, cyan; fluorine, light green; uranium, orange; chlorine, pale green; hydrogen, white. Water molecules are omitted for clarity.

MD simulations showed that the hydrophilic functional groups directed almost perpendicularly into the aqueous environment, exhibiting minimal interaction with the hydrophobic surface (Figure 3a). There is significant structuring of water molecules around the phosphoryl groups, forming a hydration sphere that includes approximately 4 water molecules. Moreover, the presence of residual fluorine atoms within the carbon lattice resulted in increased structural rigidity compared to other graphene derivatives, such as graphene acid.^56^

### 3.3 Uranium Sorption Studies of PG

**Figure 4:**
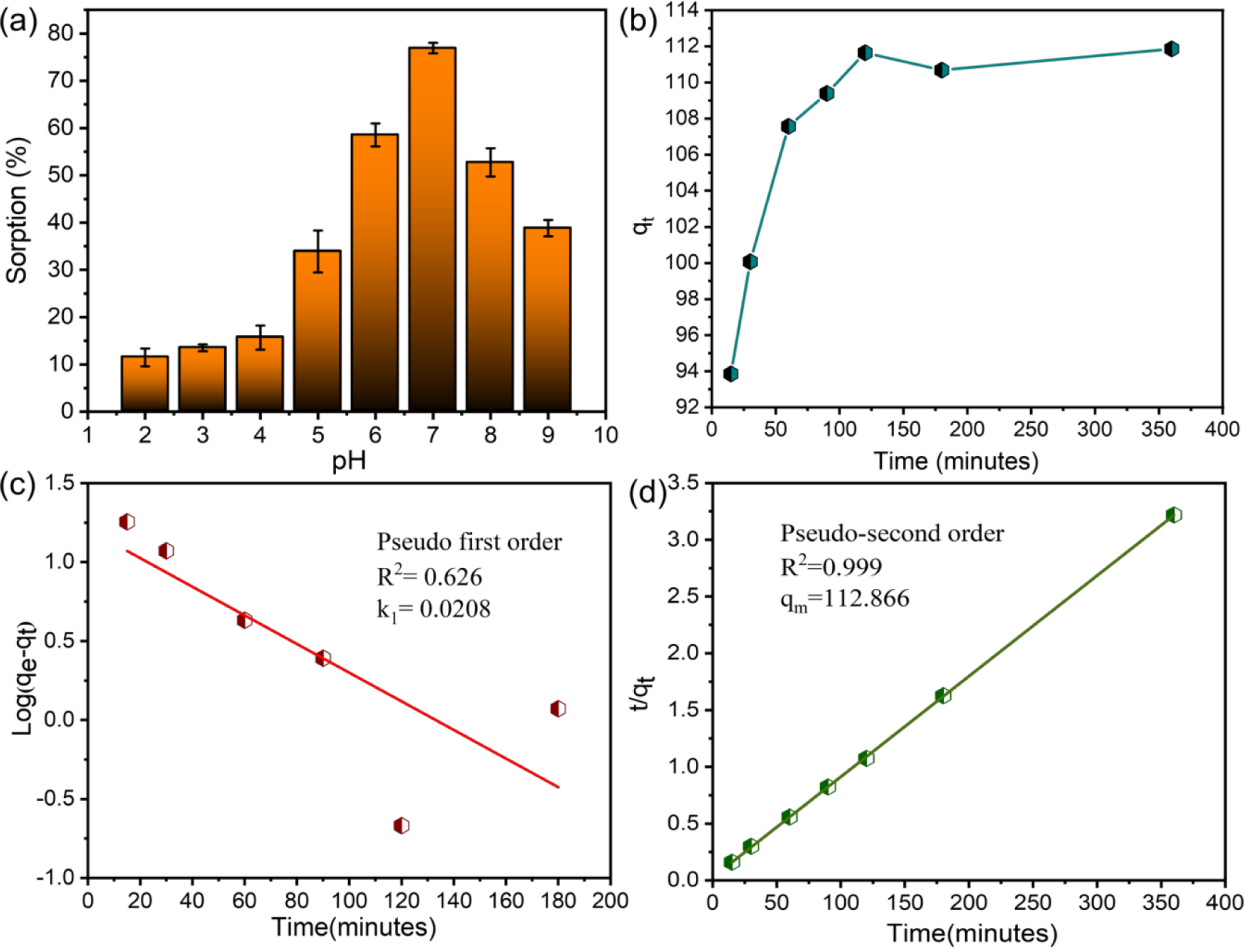
(a) Impact of pH on U(VI) sorption efficiency onto PG (initial uranium concentration = 500 mg L^–1^, equilibration time = 3 h, PG mass = 10 mg, U(VI) solution volume = 3 mL, temperature = 25 ± 1 °C); (b) influence of contact time on U(VI) sorption capacity for PG (initial concentration = 500 mg L^–1^, pH = 5, PG mass = 10 mg, U(VI) solution volume = 3 mL, temperature = 25 ± 1 °C; (c) linear plots for pseudo-first order kinetics for the adsorption of U(VI); (d) linear plots for pseudo-second order kinetics for the adsorption of U(VI).

Based on the distinctive properties of PG, we conducted uranium capture studies. U(VI) sorption onto PG is highly sensitive to solution pH. Fluctuations in pH affect the adsorption process due to their impact on both the surface charge of phosphoryl binding sites (modulated by protonation/deprotonation) and the distribution of different metal ion species.^57^ Sorption studies were conducted experimentally across a pH spectrum ranging from 2 to 9, revealing the pivotal role of pH in governing the sorption behavior of U(VI) from aqueous media. The sorption propensity rises with increasing pH, reaching a peak at pH 7, where a maximum sorption efficiency of approximately 77% is attained. MD simulations further supported that PG effectively absorbs a substantial portion of U(VI) from the solution (Figure 3c). The structure with PO_4_^–1^ groups showed a very similar value when, on average, approximately 80% of all ions in the simulated box interacted directly with the surface (Figure 3c). A decline in sorption efficiency is observed at pH 8 and 9. As indicated by MD simulations, this might be connected to the stacking arrangement of individual sheets, where the structure with PO_4_^–2^ groups was more compact and thus had less space inter channels resulting from the stacking of individual layers (Figure S2). The sorption capacity (*q*_e_) at pH 7 was quantified to be 111.9 mg g^–1,^ as shown in Figure 4a. Moreover, uranium predominantly exists in the hexavalent state as uranyl ion (UO_2_^2+^) in aqueous solutions. In acidic pH (<3) the uranyl ion UO_2_^2+^ is the dominant species. It has a high positive charge, which makes it highly susceptible to electrostatic interactions with the functional groups in PG (P=O). Also, the phosphoryl groups are protonated (P–OH^+^), reducing their ability to bind uranyl ions through coordination. Thus, adsorption efficiency decreases. Upon increasing the pH (3–6), hydrolyzed species such as UO_2_(OH)^+^ and UO_2_(OH)_2_ start to form. These species have a reduced positive charge, which lowers electrostatic attraction but can still interact with adsorbent functional groups via coordination bonds. The phosphoryl groups are deprotonated (P=O or P–O^–^), enhancing their ability to coordinate with UO_2_^2+^ or partially hydrolyzed uranium species. This corresponds to the optimal adsorption in this pH range.^6^ The kinetics of U(VI) sorption by PG in aqueous media were explored by varying the contact time and observing the resulting changes in sorption capacity. Figure 4b demonstrates the effect of contact time on the sorption capacity. Initially, the sorption capacity for U(VI) by PG increased with contact time, reaching saturation beyond 180 min, representing the optimal equilibration duration (Table S1). Additionally, this study explored the kinetics of U(VI) adsorption using both the pseudo-first-order and pseudo-second-order models. To determine the kinetic parameters, such as the rate constant (*k*), we fitted the sorption data to each model. For the pseudo-first-order model, we derived the rate constant and correlation coefficient by analyzing linear plots of log(*q*_e_ – *q*_*t*_) against time (*t*), as illustrated in Figure 4c. Conversely, for the pseudo-second-order model, we constructed plots of *t*/*q_t_* versus time (*t*), depicted in Figure 4d, to obtain the respective parameters. The experimental kinetic data were most accurately described by the pseudo-second-order model, as evidenced by a comparison of correlation coefficients (*R*^2^). The corresponding rate constants and *R*^2^ values are presented in Table S1.

Unlike simple physical adsorption, the sorption of U(VI) onto PG is predominantly driven by chemical interactions, particularly by the strong P–O···U coordination bonding. To further emphasize the role of vacancies and defects, we conducted adsorption studies on typical graphene oxide (GO), defect-engineered nitrogen-doped graphene (NG), and O-phosphorylethanolamine-based graphene (PG). The results demonstrated that PG exhibited significantly higher uranium adsorption compared to both GO and NG (Figure S3). This is evident from the observation that PG follows pseudo-second-order adsorption kinetics. The diffusion of U(VI) ions significantly impacts the sorption rate. U(VI) sorption capacity initially increases rapidly due to numerous available binding sites. As these sites saturate, the sorption rate decreases. This rate limitation primarily arises from the reduced ability of the material to accommodate additional U(VI) ions. As a result, additional ion attachment is impeded, and pores within the material become clogged. The restricted movement and diffusion of U(VI) ions, due to these hindrances, lead to the observed slower sorption rates. The influence of initial U(VI) concentration on sorption capacity was investigated in aqueous solutions at pH 7 by conducting sorption experiments across a concentration range of 100 mg L^–1^ to 900 mg L^–1^. The release of U(VI) previously adsorbed onto phosphoryl-functionalized graphene (PG) was quantified via desorption studies.

**Figure 5:**
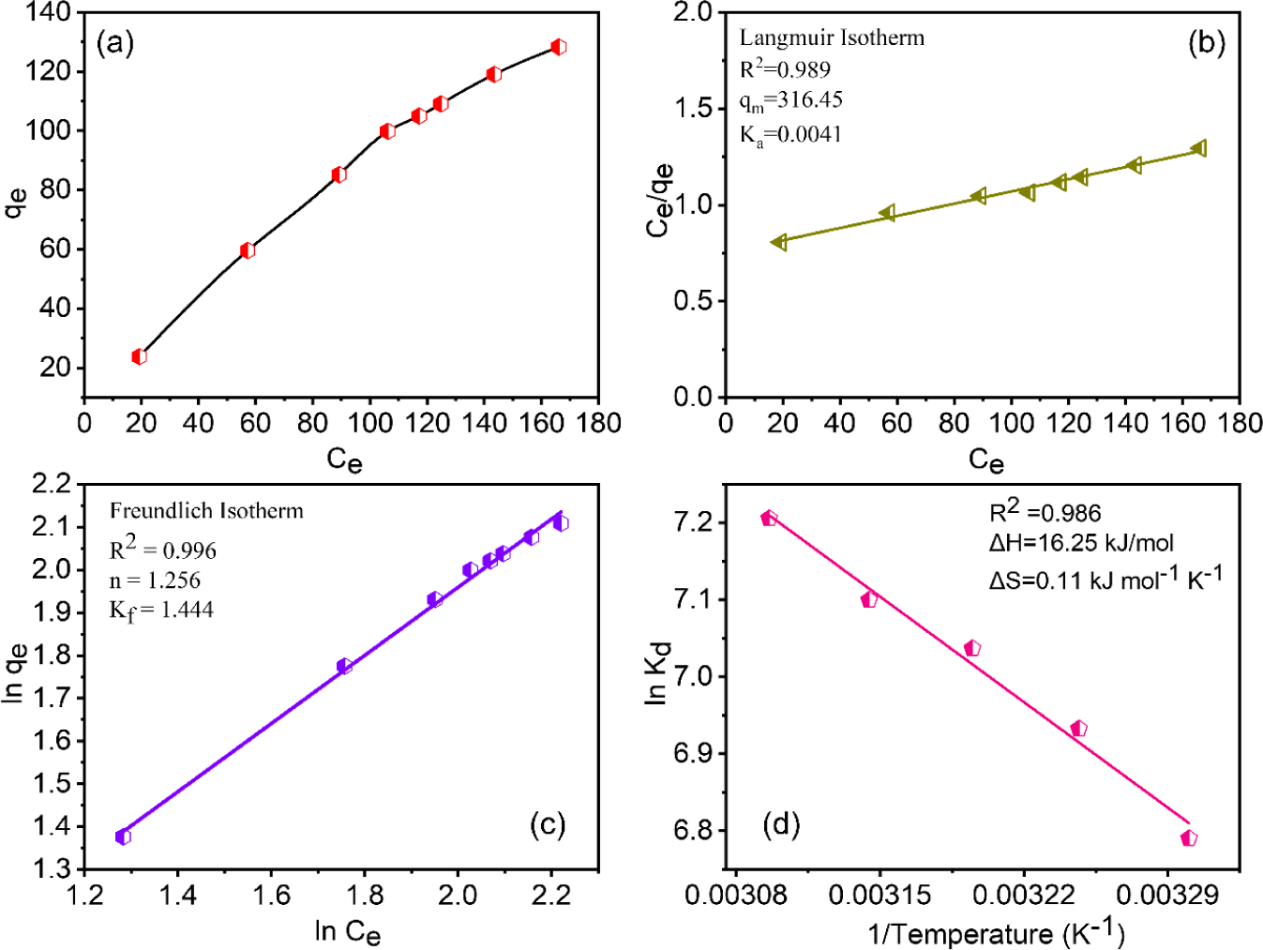
(a) Effect of U(VI) concentration on the sorption; (b) fit using linearized Langmuir isotherm for the adsorption of U(VI); (c) fit using linearized Freundlich isotherm; (d) plot of ln *K*_d_ versus 1/*T* for U(VI) adsorption on PG, initial concentration of uranium = 500 mg L^–1^.

The most effective desorbing agent identified was 0.1 M Na_2_CO_3_, which achieved a desorption efficiency of 91.76% (Figure S4a). The formation of a stable uranium carbonate complex [UO_2_(CO_3_)_3_^4–^] is accountable for the Na_2_CO_3_ solution’s remarkable eluent efficiency.^58^ Additionally, repeated U(VI) sorption tests on PG were performed to assess its recyclability (Figure S4b). The results showed a minimal decrease in sorption capacity, indicating good recyclability and stability of the material. To the best of our knowledge, the sorption capacity values of our material are comparable to those reported for other 2D materials and their hybrids, as shown in Table S3.^59−68^ Furthermore, Figure 5a demonstrates the relationship between sorption capacity (*q*_e_) and equilibrium concentration of U(VI) (*C*_e_). It is evident from the figure that there is an initial steep increase in sorption capacity at lower concentrations followed by a slower increase at higher concentrations of U(VI). Understanding of the complex dynamics of the adsorption system is essential for elucidating the interactions between U(VI) and PG. A detailed evaluation of the Langmuir and Freundlich adsorption isotherm models was performed in this study to elucidate the underlying complexities. The adsorption of U(VI) by PG was studied in detail, and the experimental data were fitted to both the Langmuir and Freundlich isotherm models, as illustrated in Figure 5b,c, respectively. The Langmuir isotherm assumes the formation of a single layer of adsorbed U(VI) on a surface comprised of identical, noninteracting binding sites, whereas the Freundlich isotherm models adsorption processes with multilayer formation on a surface with diverse binding affinities. Further experiments were carried out by expanding the concentration range to better reveal whether the adsorption is more consistent with monolayer (Langmuir) or multilayer (Freundlich) behavior (Figure S5).

Table S2 presents the isotherm parameters and correlation coefficients for both models. The correlation coefficients for both fittings indicate that Freundlich model is more applicable to explain the sorption behavior of U(VI) on PG, suggesting that the adsorption is multilayer adsorption mechanism. This is consistent with the Freundlich isotherm suitability for modeling adsorption on heterogeneous surfaces, where binding sites possess different affinities, while the Langmuir isotherm describes adsorption on uniform surfaces with a finite number of sites, all having equal energy. Furthermore, XPS survey spectra were collected for the PG material at varying concentrations of uranyl ion in solution (150, 200, 275, 300, and 380 mg L^–1^) to investigate the multilayer adsorption phenomenon.^69,70^ A steep increase in the intensity of the U 4f peak was noted at lower concentrations, which is indicative of monolayer adsorption of the uranyl ion onto the PG surface. Upon reaching a certain concentration range, a plateau was observed, suggesting saturation of monolayer adsorption. Further increases in concentration led to a slight rise in intensity, indicating multilayer adsorption, though with a smaller slope, which suggests weaker, nonspecific interactions. Additionally, during multilayer adsorption, a slight shift in binding energy was observed, reflecting changes in the chemical environment, such as uranium interacting with other uranium ions rather than the surface functional groups of PG (Figure S9).

Fitting both models may indicate a complex surface with both homogeneous and heterogeneous characteristics. Besides, to assess the influence of diffusion on the sorption rate, experiments were conducted to plot the Weber-Morris Intraparticle Diffusion Model, yielding a relatively low *R*^2^ value of 0.897 as shown in Figure S6.

**Figure 6:**
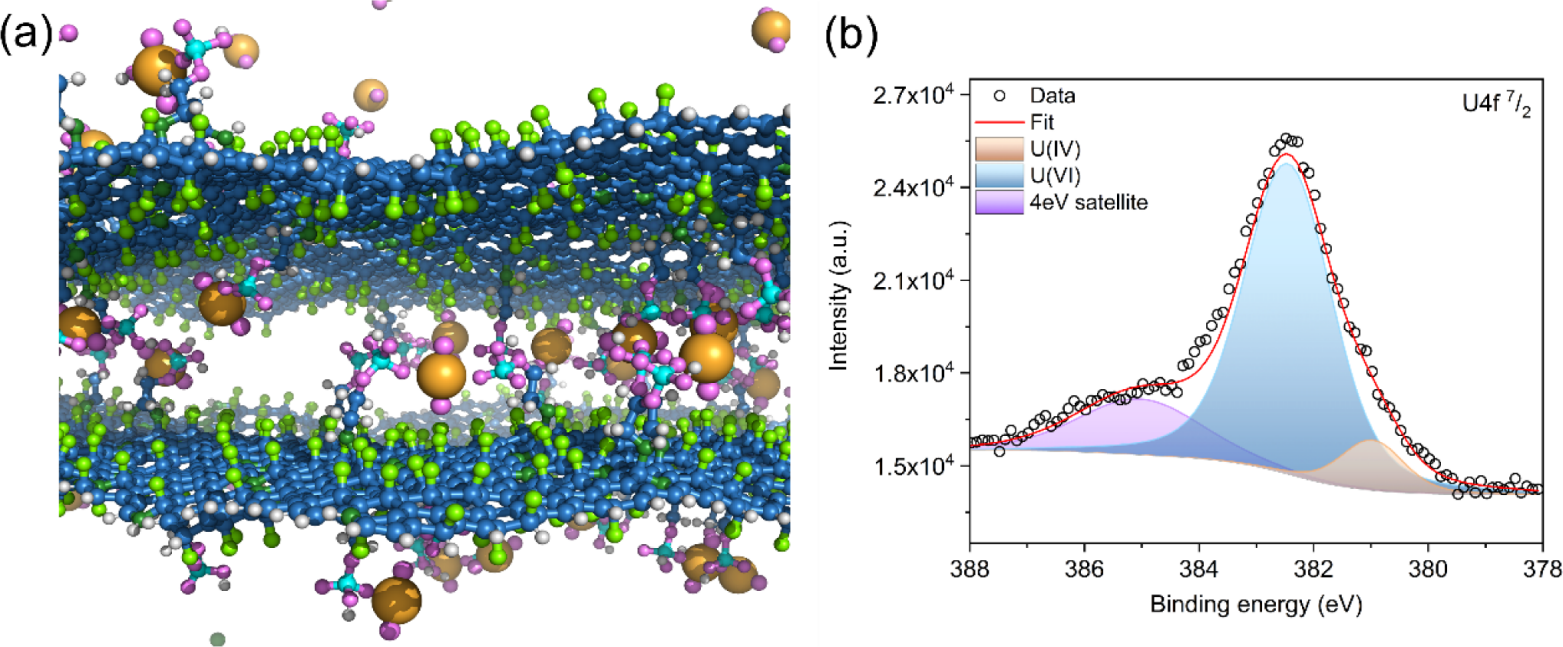
(a) Schematic illustration of uranium metal ions coordinated with phosphoryl functional groups of PG. (b) High-resolution U 4f spectra of PG. Color code: oxygen, pink; nitrogen, dark green; carbon, blue; phosphorus, cyan; fluorine, light green; uranium, orange; hydrogen, white. Water molecules are omitted for clarity.

To discern the influence of temperature on the sorption dynamics of U(VI) onto PG, a series of sorption experiments spanning temperatures from 30 to 50 °C were conducted. The relationship between ln *K*_d_ and 1/*T* (depicted in Figure 5d) yielded linear plots, from which the values of Δ*S* and Δ*H* were derived as 0.110 kJ mol^–1^ K^–1^ and 16.3 kJ mol^–1^, respectively. The positive values of Δ*H* and Δ*S* elucidate the endothermic character of the adsorption process. The thermodynamic parameters suggest a significant role of entropy in the adsorption process. The estimated Gibbs free energy change at 298 K is −16.5 kJ mol^–1^, indicating a favorable interaction between UO_2_^2+^ and the adsorbent. This favorable Δ*G* value highlights the important role of entropy in the adsorption process. The high positive entropy likely results from the hydrophobic interactions associated with the release of water molecules from the hydration spheres during adsorption. This aligns with findings from existing literature, where thermodynamic parameters have suggested an entropy-driven nature of metal adsorption on adsorbents, leading to increased sorption capacity with rising temperature. Further, to investigate the adsorption performance in a practical environment, the PG was immersed in 50 mL of simulated seawater for 3 h. The seawater had a salinity of 30 PSU and contained 7.05 ppm UO_2_^2+^ ions, along with several interfering ions: 9500 ppm of Na^+^, 1130 ppm Mg^2+,^ 360 ppm of Ca^2+,^ 350 ppm K^+^, 7 ppm of Sr^2+^, 7 ppm Fe^3+^, 7 ppm VO_4_^3–^, 8000 ppm of Cl^–,^ and 2400 ppm of SO_4_^2–^. Then concentration of UO_2_^2+^ ion was measured and calculated. Nevertheless, the phosphoryl-functionalized PG captured uranyl cations at a rate of approximately 316 mg g^–1^ in distilled water and 117.8 mg g^–1^ in simulated seawater within 5 min (Figure S7). To quantify the relative sorption of other metals on PG, an atomic absorption spectrophotometer (AAS) was used to measure Co, Mn, Zn, Cr, and Ca. The results indicate that the relative sorption of these metals is significantly lower compared to uranium on the PG surface (Table S3). The significant sorption behavior of PG can be attributed mostly to phosphoryl-functionalized groups (Scheme 1 and Figure 6a). The phosphoryl groups on the PG surface exhibit favorable coordination with U(VI), as evidenced by MD simulations (Figure 3). Furthermore, XPS analysis was employed to provide structural organization and the nature of the coordination, revealing the interaction mechanism between phosphoryl functional groups of the PG and U(VI) at the molecular level (Figure 6b). Further XPS characterization after uranium capture provided additional insights (Figures 6b and S8). As shown in the high-resolution spectrum, the U(VI) contribution U 4f 7/2 peak remained at around 382.8 eV (fully comparable with the pristine uranyl salt, Figure S8).^71^ The contribution of the U(IV) is caused by the U(VI) being reduced with the X-rays during measurement. The fact that binding energy remained unchanged can indicate that the uranyl group is adsorbed in the same coordination environment and no change of oxidation state took place.^72^

## 4 Conclusions

In summary, we have synthesized phosphoryl-functionalized graphene (PG) using fluorographene chemistry. The solvothermal synthesis process, employing a fluorinated precursor and phosphorylethanolamine, resulted in the creation of PG with distinct structural characteristics, such as reactive phosphoryl functional groups. The resultant PG exhibited remarkable sorption capacity in a pH medium, owing to its organized interlayer channels, defects within its 2D structure, and reactive phosphoryl groups. The sorption efficiency was approximately 77% from pH ∼ 7 medium, surpassing that of other sorption materials. The bonding between U(VI) and PG was confirmed by X-ray photoelectron spectroscopy (XPS), and MD simulations provide further structural insights. The findings of this research offer promising directions for the development and production of functional graphene-based materials for the selective extraction of radioactive elements from seawater and nuclear wastewater. The approach demonstrated here has the potential to deliver superior performance across a range of radioactive element extraction applications.

## Supporting Information Available

Characterization data for FG and PG using various techniques, including XRD, FT-IR, Raman, SEM, and XPS; snapshots from MD simulations and stability measurements, with a table comparing our values to those reported for similar materials in the literature (PDF)

## Notes

The raw data can be accessed at ZENODO repository: DOI: 10.5281/zenodo.14852789.
