# Supplementary material for: Phosphoryl-Graphene for High-Efficiency Uranium Separation and Recycling
Source: ACS Appl Mater Interfaces. 2025 Feb 20;17(11):17284–94. doi: 10.1021/acsami.4c22385 (PMC11931496; doi:10.1021/acsami.4c22385)
Supplement: Supplementary file 1 — am4c22385_si_001.pdf [file am4c22385_si_001.pdf]

Supporting Information

Phosphoryl-Graphene for High-Efficiency Uranium Separation and Recycling

Martin Pykal[1‡], Veronika Šedajová[1‡], Aditya Thakur[2‡], Somnath Sengupta[3], Cherukuri Venkata Siva Brahmananda Rao[3,4], Radek Zboril[1,5], Balija Sreenivasulu[2,5]*, Michal Otyepka[1,6]* and Kolleboyina Jayaramulu[2,4]*

[1]Regional Centre of Advanced Technologies and Materials, Czech Advanced Technology and Research Institute (CATRIN), Palacký University Olomouc, Šlechtitelů 27, 783 71 Olomouc, Czech Republic

[2]Hybrid Porous Materials Laboratory, Department of Chemistry, Indian Institute of Technology Jammu, Jammu & Kashmir, 181221, India.

[3]Fuel Chemistry Division, Materials Chemistry and Metal Fuel Cycle Group, Indira Gandhi Centre for Atomic Research, Kalpakkam 603102, India

[4]Homi Bhabha National Institute, Anushaktinagar, Mumbai 400094, India

[5]Nanotechnology Centre, Centre for Energy and Environmental Technologies, VŠB − Technical University of Ostrava, 17. Listopadu, Ostrava-Poruba, 708 00, Czech Republic

[6]IT4Innovations, VŠB-Technical University of Ostrava, 17. listopadu 2172/15, 70800 Ostrava-Poruba, Czech Republic

[‡]M. P., V. Š. and A.T contributed equally to this paper.

Email(s): bsrinu@igcar.gov.in, michal.otyepka@upol.cz, jayaramulu.kolleboyina@iitjammu.ac.in.

Keywords: Two-dimensional (2D) materials, phosphoryl-functionalized graphene, graphene derivatives, uranium adsorption, molecular dynamics simulations.

1. Experimental Section

1.1 Characterization Techniques

XRD measurements were performed using an X'Pert PRO MPD diffractometer (PANalytical) in the Bragg–Brentano geometry equipped with a Co X-ray tube. Samples were placed on a zero-background Si slide, gently pressed, and scanned with a step size of 0.0334°, and the 2θ range of 5°–120° was used to record the pattern. Infra-red spectra were acquired using an iS5 FTIR spectrometer (Thermo Nicolet) with the Smart Orbit ATR accessory featuring a ZnSe crystal. A drop of sample dispersion in ethanol or water was placed on a ZnSe crystal and left to dry, forming a film in ambient conditions, and spectra were recorded by summing 50 scans. Nitrogen gas flowed through the ATR accessory during background and sample measurements, and ATR and baseline correction were applied for spectrum processing. X-ray photoelectron spectroscopy (XPS) was conducted using a PHI VersaProbe II (Physical Electronics) and Nexsa G2 (Thermo Fisher) spectrometers with an Al Kα source. Transmission electron microscopy (TEM) images were obtained using a JEOL 2100 TEM with an emission gun of LaB_6 type operating at 160 kV. High-resolution TEM images were obtained using an FEI Titan electron microscope operating at 80 kV. Scanning electron microscopy (SEM) was performed using a Hitachi SU6600 instrument with an accelerating voltage of 5 kV. For these analyses, a small droplet of a material dispersion in ultrapure water (concentration approximately 0.1 mg ml^{-1}) was placed on a carbon-coated copper grid and left to dry. Raman spectra were obtained on a DXR Raman microscope using the 633 nm excitation line diode laser. The nitrogen adsorption/desorption isotherms at 77 K were measured by using 3Flex instrument (Micromeritics Instruments, Norcross, GA, USA). Before the nitrogen adsorption/desorption experiments, the GN samples were degassed at 100 ºC for 16 h under vacuum.

1.2 U(VI) sorption studies

The sorption efficiency (%), amount of metal ion sorbed onto PG (q_e; mg g−1) and distribution coefficient (K_d) for U(VI) by PG were calculated using the following equations:

$$\text{Sorption efficiency}\,(\%) = \frac{(C_o - C_e)}{C_o} \times 100 \tag{S1}$$

$$q_e = \frac{(C_o - C_e) \times V}{m} \tag{S2}$$

$$K_d = \frac{(C_o - C_e)}{C_e} \times \frac{V}{m} \tag{S3}$$

Here, C_o and C_e denote the initial and equilibrium concentrations (mg L^{-1}) of U(VI) ions, V is the volume of solution (L) added, and m is the mass of sorbents PG (g).

1.3 Kinetics of Adsorption

The following equations describe the amount of adsorbate that accumulates on the surface of the adsorbent over time:

Pseudo-first order:

$$log(q_e - q_t) = \log q_e - \frac{k_1}{2.303}t \quad (S4)$$

Pseudo-second order:

$$\frac{t}{q_t} = \frac{1}{k_2 q_e^2} + \frac{t}{q_e} \quad (S5)$$

Here, q_e represents the amount (mg) of U(VI) adsorbed onto the PG (g) at equilibrium, while q_t refers to the amount at time t. k_1 and k_2 are the rate constants corresponding to the pseudo-first order and pseudo-second order kinetics, respectively.

1.4 Adsorption isotherms

The Langmuir and Freundlich models are represented by the following equations:

Langmuir:

$$\frac{C_e}{q_e} = \frac{1}{K_a q_m} + \frac{C_e}{q_m} \quad (S6)$$

Freundlich:

$$ln\, q_e = \ln K_f + \frac{1}{n}\ln C_e \quad (S7)$$

where, q_e is the sorption capacity at equilibrium (mg g^{-1}), C_e is the equilibrium concentration (mg L^{-1}), and q_m is the maximum sorption capacity (mg g^{-1}), K_a is the Langmuir constant (L g^{-1}) and K_f is the Freundlich constant (mg g^{-1}) (L $mg^{-1})^{1/n}$, respectively, and 1/n is the Freundlich's heterogeneity coefficient.

1.5 Thermodynamic parameters

The thermodynamic parameters (ΔH, ΔS, and ΔG) for the adsorption of the U(VI) on the PG were calculated at different temperatures using the following equations:

$$ln\, K_d = \frac{\Delta S}{R} - \frac{\Delta H}{RT} \quad (S8)$$

$$\Delta G = \Delta H - T\Delta S \quad (S9)$$

The values of ΔH and ΔS can be derived from the slope and intercept obtained from the van't Hoff linear plot between ln K_d and 1/T.

Table S1. Kinetics parameters for the adsorption of U(VI) on PG.

Pseudo-first order			Pseudo-second order		
k (min^{-1})	R^2	q_e (mg g^{-1})	k (g mg^{-1} min^{-1})	R^2	q_e (mg g^{-1})
0.0208	0.626	16.106	0.003019	0.999	112.866

Table S2. The isotherm parameters and correlation coefficients for both Langmuir and Freundlich model.

**Model**	**Parameters**		
	$\mathbf{q_{max}}$	$\mathbf{K_L}$	$\mathbf{R^2}$
**Langmuir**	218.81	0.0072	0.971
	**n**	$\mathbf{K_f}$	$\mathbf{R^2}$
**Freundlich**	1.468	2.19	0.993

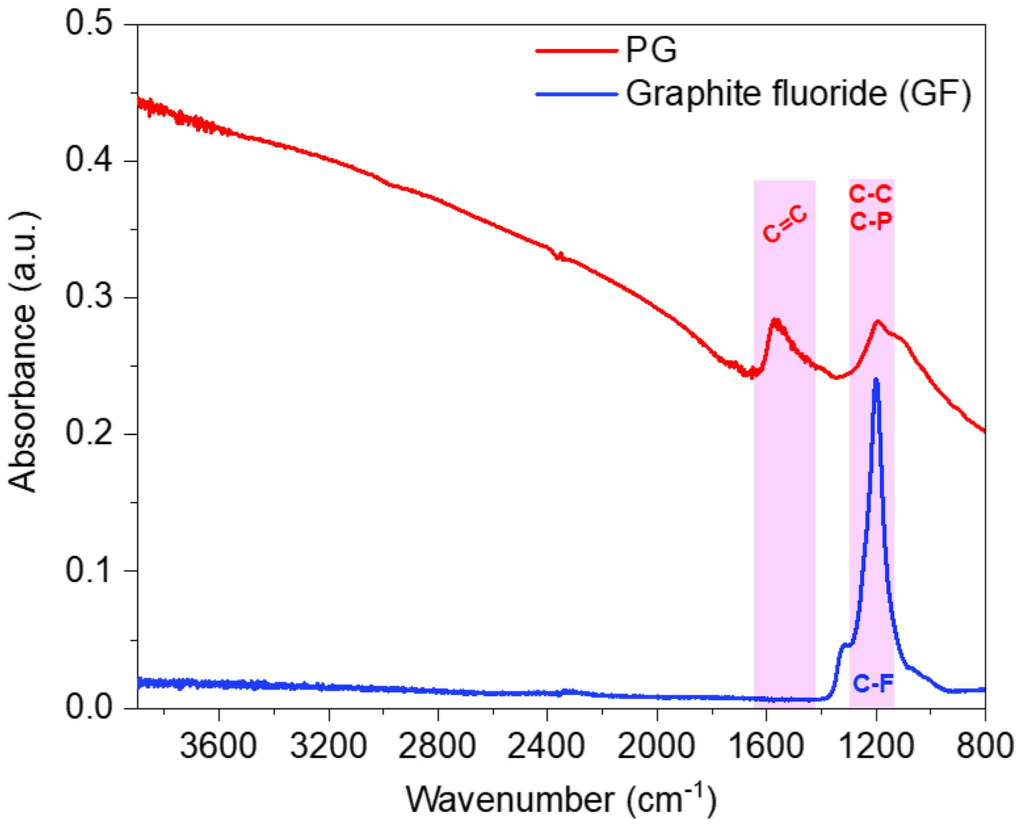

Figure S1. FT-IR Spectra of FG (bottom) and PG (top).

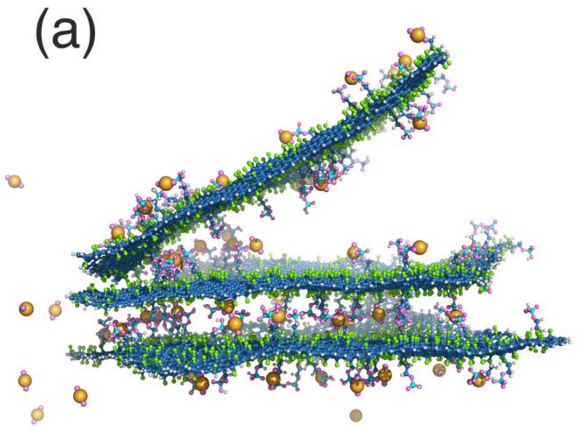

Figure S2. Snapshots from MD simulations showing the stacking arrangement of PG sheets depending on the protonation of the phosphoryl (a) PO_4^{-1} and (b) PO_4^{-2} groups. Water molecules and ions are omitted for clarity.

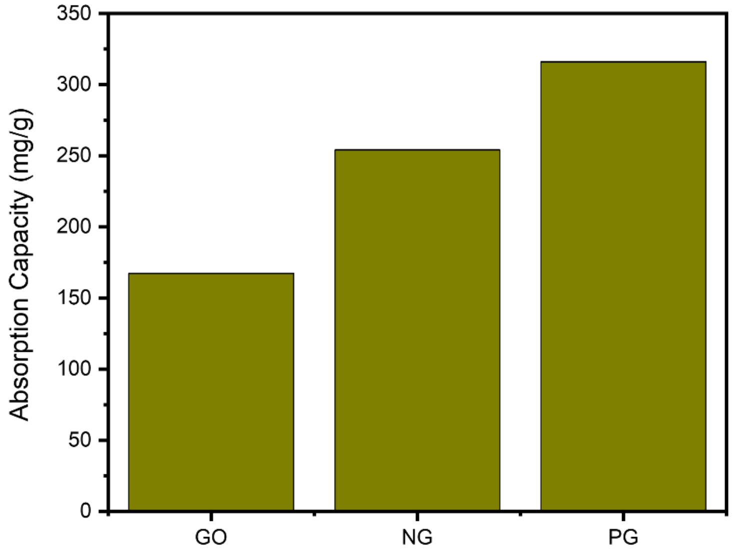

Figure S3. Maximum uranium adsorption studies of various graphene derivatives, including pristine graphene oxide (GO), fluorographene-derived nitrogen-doped graphene (NG), and phosphoryl-functionalized graphene (PG) at pH ~7.

Discussion:

Uranium adsorption studies were conducted on typical graphene oxide (GO), nitrogen-doped graphene (NG), and O-phosphorylethanolamine-based graphene (PG). However, pristine fluorographene (FG) exhibits superhydrophobic properties, which make it challenging to measure uranium adsorption in aqueous environments. The results demonstrated that PG exhibited significantly higher uranium adsorption compared to both GO and NG. This finding underscores the importance of phosphoryl groups, in conjunction with nitrogen doping within the vacancies and defects, in enhancing the material's uranium adsorption capacity.

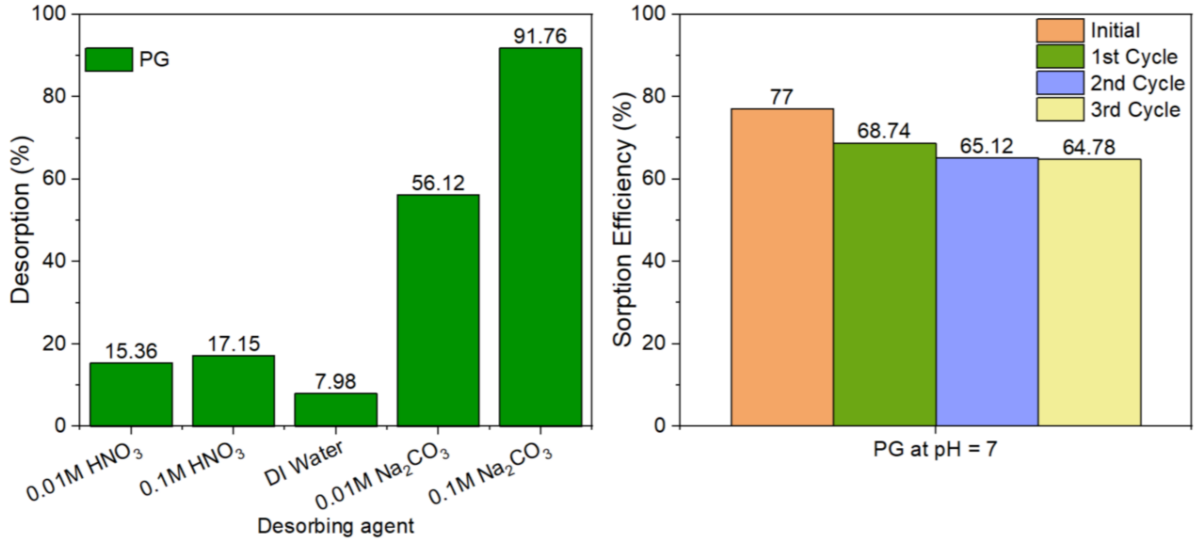

Figure S4. (a) Desorption (%) of PG using different desorbing agents; (b) Recyclability studies plot of PG.

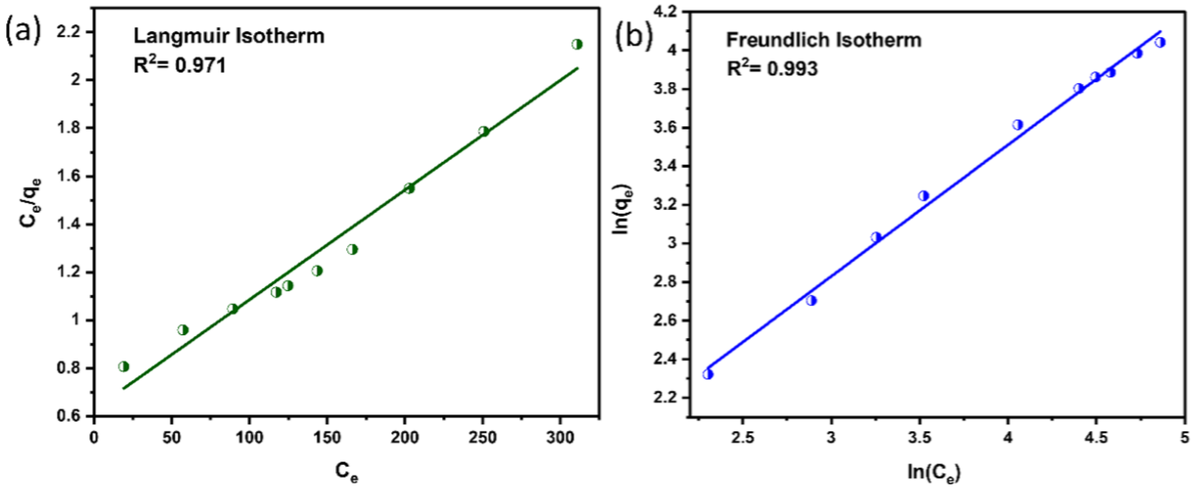

Figure S5. (a) Fit using linearized Langmuir isotherm for the adsorption of U(VI); (b) Fit using linearized Freundlich isotherm (concentration range: 10 mg L^{-1} to 400 mg L^{-1}).

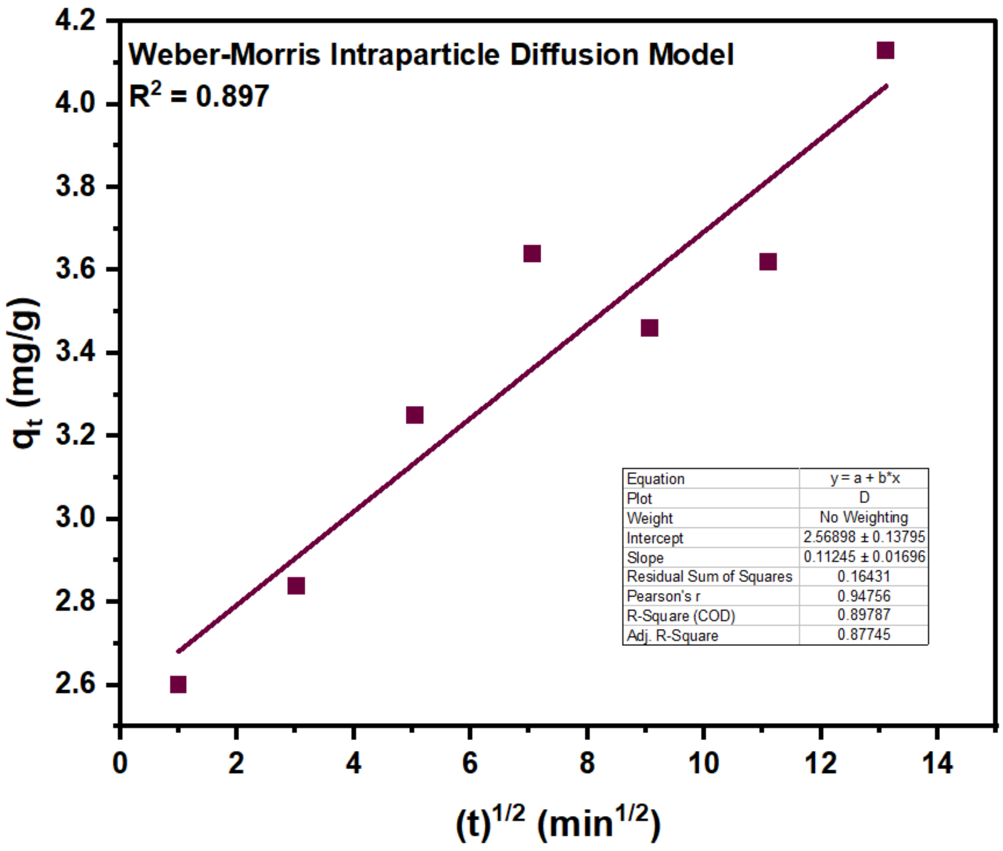

Figure S6. Weber-Morris intraparticle diffusion model plot illustrating the influence of diffusion on the sorption rate, with a relatively low R^2 value of 0.897.

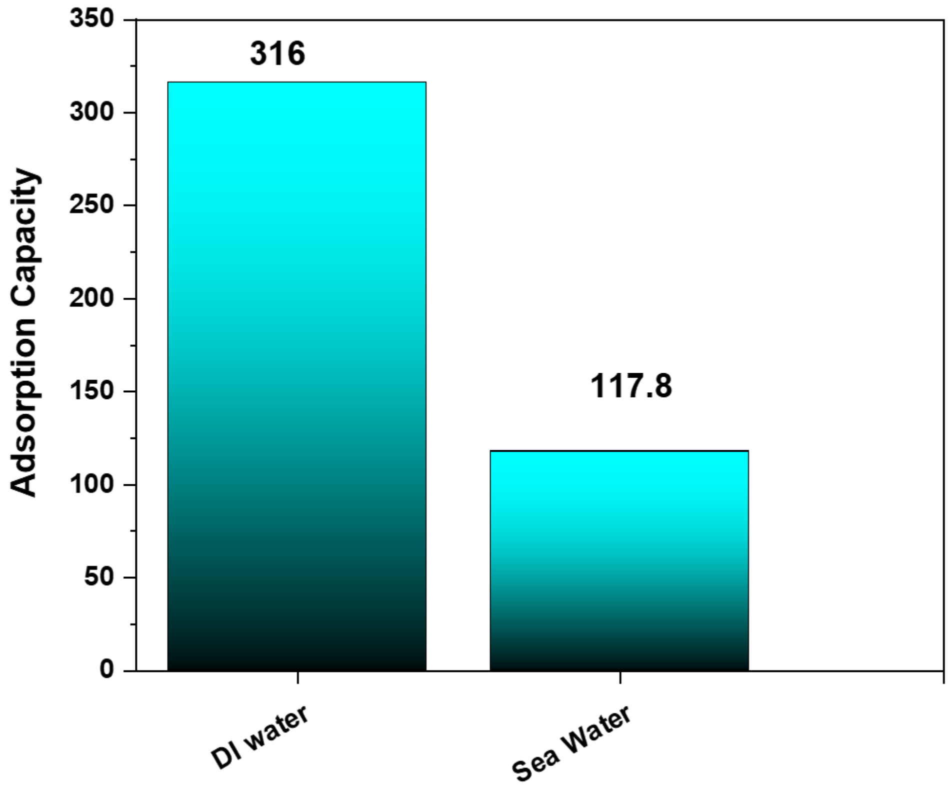

Figure S7. Change in adsorption capacity parameter from DI water to stimulated sea water of PG.

Discussion:

We have conducted competitive adsorption studies to evaluate the selectivity of phosphoryl-functionalized graphene (PG) for uranium. These studies were performed using both distilled water and artificial seawater, where a mixture of competing salts (KCl, NaCl, $CaCl_2$, $Mg(NO_3)_2$, $SrCl_2$, $CdCl_2$) was added to the uranium solution. To thoroughly investigate the adsorption performance in practical environment, the PG was further examined in the 50 mL of simulated seawater (salinity of 30) with 7.05 ppm UO_2^{2+} ion and interfering ions (including 9500 ppm Na^+, 1130 ppm Mg^{2+}, 360 ppm Ca^{2+}, 350 ppm K^+, 7 ppm Sr^{2+}, 7 ppm Fe^{3+}, 7 ppm VO^{3-}, 8000 ppm Cl^-, and 2400 ppm SO_4^{2-}) for 3 hours. Then concentration of UO_2^{2+} ion was measured and calculated. PG captured approximately 316 mg g^{-1} and 117.8 mg g^{-1} of uranyl cations within 5 minutes in distilled water and seawater, respectively. The results from these studies further support the selective capture of uranium by PG from complex aqueous matrices.

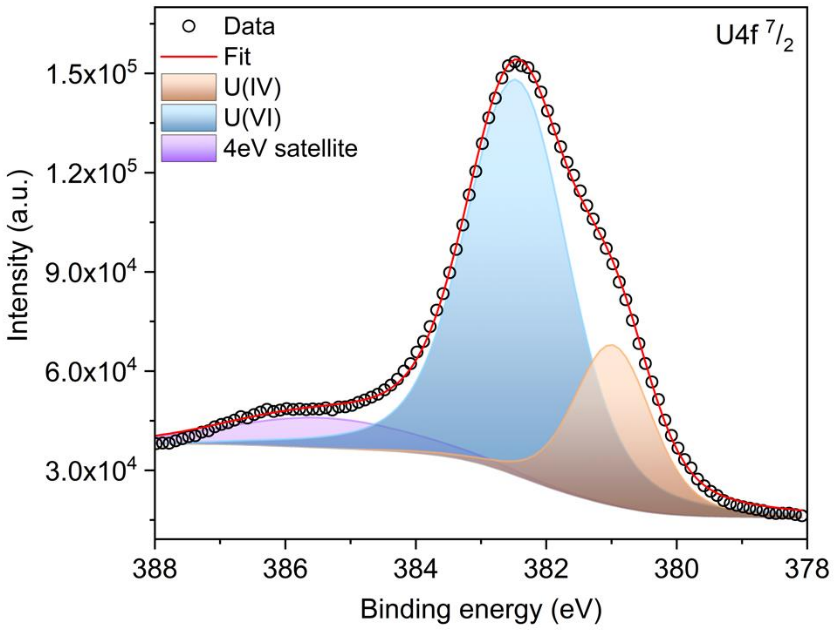

Figure S8. High resolution XPS spectra of U4f of pristine uranium salt.

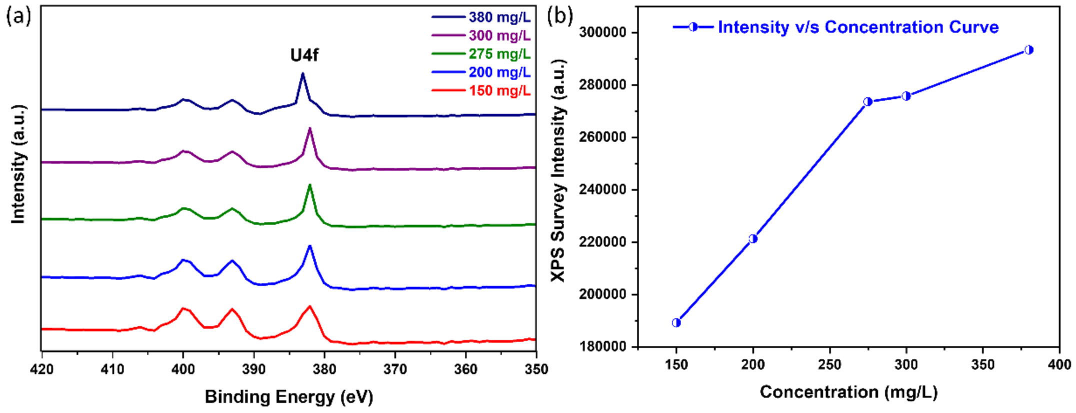

Figure S9. (a) XPS survey spectra showing the relative intensity of U4f peak at varying concentrations; (b) Plot between XPS survey spectra intensity versus concentration.

Table S3. Quantification of other metal sorption on the surface of the PG.

Metal	Initial Concentration ($mg\ L^{-1}$)	Concentration after Adsorption on PG surface ($mg\ L^{-1}$)
Cu	2	1.74
Mn	0.5	0.15
Zn	2	1.67
Cr	1	0.68
Ca	25	20.36

Table S4. Summary of uranium adsorption performance of graphene-based materials from literature.

S.No.	Adsorbents	Adsorption Capacity ($mg\ g^{-1}$)	Freundlich constant ($mg\ g^{-1}$) ($L\ mg^{-1}$)$^{1/n}$	Recyclability	Stability	pH	Reference
1	GO-NH2	215.2	93.89	90%	3 cycles	5.5	[1]
2	CGO	240.1	49.64	-	-	~4.5	[2]
3	GO-ACF	298.0	215.4	-	-	5.5	[3]
4	AO-MGO	284.9	158.6	-	-	5	[4]
5	GO	208.3	48.25	-	-	4	[5]
6	PAM/GO	166.1	0.424 ($mmol^{1-n}$ $L^n g^{-1}$)	-	-	6	[6]
7	rGO-ZnO	256.4	11.863	-	-	5.5	[7]
8	MIL-68/GO	375.0	-	240 $mg\ g^{-1}$	5 cycles	8	[8]
9	GO-CS	384.6	-	93.1%	3 cycles	8.3	[9]
10	Sulfonated graphene oxide	310	84.85	-	-	6	[10]
11	Phosphate-functionalized graphene oxide	252	21.8 (mol^{1-n} $L^n g^{-1}$)	-	-	4	[11]
12	Ethylenediaminetetraacetic acid-graphene oxide	213	79.49	173.53 $mg\ g^{-1}$	4 cycles	6	[12]
13	Halloysite@graphene oxide	161	6.63 ($mmol^{1-n}$ $L^n g^{-1}$)	-	-	7	[13]
14	Graphene oxide/ polydopamine	145	30.84	>90%	6 cycles	4	[14]
15	Amino-functionalized magnetic graphene oxide	141	103 (mg^{1-n} $L^n g^{-1}$)	123.5 $mg\ g^{-1}$	4 cycles	6	[15]
16	Nickel ferrite /graphene oxide	135	31.75	96.5%	5 cycles	5	[16]
17	Magnetic cucurbit[6]uril/graphene oxide	123	31.04	90.58%	5 cycles	5	[17]
18	MXene/PAO hydrogel beads	625	-	90%	5 cycles	7	[18]
19	V_2CT_x	174	29.93 $mg\ g^{-1}$	-	-	5	[19]
20	PG	316.5	2.19	91.76 %	4 cycles	7	This work

References:

1. Liu, S.; Li, S.; Zhang, H.; Wu, L.; Sun, L.; Ma, J. Removal of Uranium (VI) from Aqueous Solution using Graphene Oxide and its Amine-Functionalized Composite. *Journal of Radioanalytical and Nuclear Chemistry* **2016**, 309, 607.
2. Wang, Z.; Wang, Y.; Liao, J.; Yang, Y.; Liu, N.; Tang, J. Improving the Adsorption Ability of Graphene Sheets to Uranium through Chemical Oxidation, Electrolysis and Ball-Milling. *Journal of Radioanalytical and Nuclear Chemistry* **2016**, 308, 1095.
3. Chen, S.; Hong, J.; Yang, H.; Yang, J. Adsorption of Uranium (VI) from Aqueous Solution using a Novel Graphene Oxide-Activated Carbon Felt Composite. *Journal of Environmental Radioactivity* **2013**, 126, 253.
4. Zhao, Y.; Li, J.; Zhang, S.; Chen, H.; Shao, D. Efficient Enrichment of Uranium (VI) on Amidoximated Magnetite/Graphene Oxide Composites. *RSC advances* **2013**, 3 (41), 18952.
5. Ding, C.; Cheng, W.; Sun, Y.; Wang, X. Retracted Article: Determination of Chemical Affinity of Graphene Oxide Nanosheets with Radionuclides Investigated by Macroscopic, Spectroscopic and Modeling Techniques. *Dalton Transactions* **2014**, 43 (10), 3888.
6. Song, W.; Wang, X.; Wang, Q.; Shao, D.; Wang, X. Plasma-Induced Grafting of Polyacrylamide on Graphene Oxide Nanosheets for Simultaneous Removal of Radionuclides. *Physical Chemistry Chemical Physics* **2015**, 17 (1), 398.
7. Kaptanoglu, I. G.; Yusan, S. Adsorption of Uranium Ions from Aqueous Solutions by Graphene-Based Zinc Oxide Nanocomposites. *Journal of Radioanalytical and Nuclear Chemistry* **2023**, 332 (11), 4705.
8. Zhu, J.; Zhang, H.; Liu, Q.; Wang, C.; Sun, Z.; Li, R.; Liu, P.; Zhang, M.; Wang, J. Metal-Organic Frameworks (MIL-68) Decorated Graphene Oxide for Highly Efficient Enrichment of Uranium. *Journal of the Taiwan Institute of Chemical Engineers* **2019**, 99, 45.
9. Huang, Z.; Li, Z.; Zheng, L.; Zhou, L.; Chai, Z.; Wang, X.; Shi, W. Interaction Mechanism of Uranium (VI) with Three-Dimensional Graphene Oxide-Chitosan Composite: Insights from Batch Experiments, IR, XPS, and EXAFS Spectroscopy. *Chemical Engineering Journal* **2017**, 328, 1066.
10. Zhang, Z.-b.; Qiu, Y.-f.; Dai, Y.; Wang, P.-f.; Gao, B.; Dong, Z.-m.; Cao, X.-h.; Liu, Y.-h.; Le, Z.-g., Synthesis and Application of Sulfonated Graphene Oxide for the Adsorption of Uranium(VI) from Aqueous Solutions. *Journal of Radioanalytical and Nuclear Chemistry* **2016**, 310 (2), 547-557.
11. Liu, X.; Li, J.; Wang, X.; Chen, C.; Wang, X., High Performance of Phosphate-Functionalized Graphene Oxide for the Selective Adsorption of U(VI) from Acidic Solution. *Journal of Nuclear Materials* **2015,** *466*, 56-64.
12. Liu, S.; Zhang, H.; Peng, D.; Yuan, D.; Wu, L.; Ma, J., Uranium Uptake with Graphene Oxide Sponge Prepared by Facile EDTA-Assisted Hydrothermal Process. *International Journal of Energy Research* **2017,** *41* (2), 263-273.
13. Xiao, J.; Xie, S.; Jing, Y.; Yao, Y.; Wang, X.; Jia, Y., Preparation of Halloysite@Graphene Oxide Composite and its Application for High-Efficient Decontamination of U(VI) from Aqueous Solution. *Journal of Molecular Liquids* **2016,** *220*, 304-310.
14. Zhao, Z.; Li, J.; Wen, T.; Shen, C.; Wang, X.; Xu, A., Surface Functionalization Graphene Oxide by Polydopamine for High Affinity of Radionuclides. *Colloids and Surfaces A: Physicochemical and Engineering Aspects* **2015,** *482*, 258-266.
15. Chen, L.; Zhao, D.; Chen, S.; Wang, X.; Chen, C., One-Step Fabrication of Amino Functionalized Magnetic Graphene Oxide Composite for Uranium(VI) Removal. *Journal of Colloid and Interface Science* **2016,** *472*, 99-107.
16. Lingamdinne, L. P.; Choi, Y.-L.; Kim, I.-S.; Yang, J.-K.; Koduru, J. R.; Chang, Y.-Y., Preparation and Characterization of Porous Reduced Graphene Oxide Based Inverse Spinel

Nickel Ferrite Nanocomposite for Adsorption Removal of Radionuclides. *Journal of Hazardous Materials* **2017**, 326, 145-156.
17. Shao, L.; Wang, X.; Ren, Y.; Wang, S.; Zhong, J.; Chu, M.; Tang, H.; Luo, L.; Xie, D., Facile Fabrication of Magnetic Cucurbit[6]uril/Graphene Oxide Composite and Application for Uranium Removal. *Chemical Engineering Journal* **2016**, 286, 311-319.
18. Zhou, Y.; Yang, J.; Zhou, N.; Hao, H.; Jiang, X.; Lei, F.; Shi, K.; Zhao, Y.; Zhou, G.; Liu, T.; Xing, S., Amidoxime-Functionalized MXene Beads for the Effective Capture of Uranium from Wastewater with High Fluoride Concentrations. *Chemical Engineering Journal* **2023**, 471, 144647.
19. Wang, L.; Yuan, L.; Chen, K.; Zhang, Y.; Deng, Q.; Du, S.; Huang, Q.; Zheng, L.; Zhang, J.; Chai, Z.; Barsoum, M. W.; Wang, X.; Shi, W., Loading Actinides in Multilayered Structures for Nuclear Waste Treatment: The First Case Study of Uranium Capture with Vanadium Carbide MXene. *ACS Applied Materials & Interfaces* **2016**, 8 (25), 16396-16403.
